# Yeast *IME2* Functions Early in Meiosis Upstream of Cell Cycle-Regulated SBF and MBF Targets

**DOI:** 10.1371/journal.pone.0031575

**Published:** 2012-02-29

**Authors:** George S. Brush, Nicole A. Najor, Alan A. Dombkowski, Daniela Cukovic, Kara E. Sawarynski

**Affiliations:** 1 Department of Oncology, Wayne State University School of Medicine, Detroit, Michigan, United States of America; 2 Program in Molecular Biology & Genetics, Barbara Ann Karmanos Cancer Institute, Detroit, Michigan, United States of America; 3 Cancer Biology Graduate Program, Wayne State University School of Medicine, Detroit, Michigan, United States of America; 4 Department of Pharmacology, Wayne State University School of Medicine, Detroit, Michigan, United States of America; 5 Division of Clinical Pharmacology & Toxicology, Department of Pediatrics, Wayne State University School of Medicine, Children's Hospital of Michigan, Detroit, Michigan, United States of America; National Cancer Institute, United States of America

## Abstract

**Background:**

In *Saccharomyces cerevisiae*, the G1 cyclin/cyclin-dependent kinase (CDK) complexes Cln1,-2,-3/Cdk1 promote S phase entry during the mitotic cell cycle but do not function during meiosis. It has been proposed that the meiosis-specific protein kinase Ime2, which is required for normal timing of pre-meiotic DNA replication, is equivalent to Cln1,-2/Cdk1. These two CDK complexes directly catalyze phosphorylation of the B-type cyclin/CDK inhibitor Sic1 during the cell cycle to enable its destruction. As a result, Clb5,-6/Cdk1 become activated and facilitate initiation of DNA replication. While Ime2 is required for Sic1 destruction during meiosis, evidence now suggests that Ime2 does not directly catalyze Sic1 phosphorylation to target it for destabilization as Cln1,-2/Cdk1 do during the cell cycle.

**Methodology/Principal Findings:**

We demonstrated that Sic1 is eventually degraded in meiotic cells lacking the *IME2* gene (*ime2Δ*), supporting an indirect role of Ime2 in Sic1 destruction. We further examined global RNA expression comparing wild type and *ime2Δ* cells. Analysis of these expression data has provided evidence that Ime2 is required early in meiosis for normal transcription of many genes that are also periodically expressed during late G1 of the cell cycle.

**Conclusions/Significance:**

Our results place Ime2 at a position in the early meiotic pathway that lies upstream of the position occupied by Cln1,-2/Cdk1 in the analogous cell cycle pathway. Thus, Ime2 may functionally resemble Cln3/Cdk1 in promoting S phase entry, or it could play a role even further upstream in the corresponding meiotic cascade.

## Introduction

Gametogenesis includes the specialized process of meiosis whereby haploid cells are generated from diploid precursors. This reduction in ploidy is achieved through one round of DNA replication during “pre-meiotic” S phase followed by two successive rounds of chromosome segregation during the meiotic divisions. As in the mitotic cell cycle, commitment to DNA replication in meiosis involves a highly orchestrated sequence of events to ensure that the genome is efficiently and accurately duplicated. While considerable insight into the regulatory processes that control S phase entry during the cell cycle has been elucidated, the analogous meiotic process has not been clearly defined.

The budding yeast *Saccharomyces cerevisiae* has been an invaluable model for characterizing fundamental cell cycle processes, including those that govern S phase entry. This system has also contributed greatly to our understanding of meiosis, which is linked to sporulation in *S. cerevisiae*. Based on our current knowledge, cell cycle and meiotic events that immediately precede initiation of DNA replication in *S. cerevisiae* appear to be conserved. Focusing specifically on CDK, both processes require the B-type cyclin/CDK complexes Clb5,-6/Cdk1, which in the cell cycle and meiosis are rendered active through destruction of the B-type cyclin/CDK inhibitor Sic1 [1]–[4]. Recently, we have shown that B-type cyclin/CDK activities, presumably Clb5,-6/Cdk1-mediated, function to prevent re-initiation of DNA replication after normal origin firing during meiosis [5] as they do during the cell cycle [6]. While the mechanisms by which these Cdk1 activities operate to control DNA replication during meiosis have not been characterized, it is likely that they function as they do during the cell cycle by catalyzing phosphorylation of various DNA replication proteins to regulate activation of the MCM replicative helicase (see [7]). In addition to CDK, the Dbf4-dependent Cdc7 protein kinase (DDK) is required for proper initiation of DNA replication during both the cell cycle and meiosis [8]–[10]. The key target of DDK during the cell cycle is the MCM complex [7], but, as in the case of CDK, the DDK mechanism during meiosis has not been well defined.

In contrast to the processes that directly impinge on replication origin firing and prevention of inappropriate re-firing, the upstream regulatory events that set these mechanisms into motion during the cell cycle and meiosis are considerably different. G1 cyclin/CDK complexes coordinate progression from G1 to S phase during the cell cycle. Cln3/Cdk1 can be considered the apical kinase in this pathway, as its activity in late G1 leads to transcription of genes that control S phase progression and DNA replication [11]–[13]. One mechanism by which Cln3/Cdk1 achieves this upregulation is by catalyzing phosphorylation of the transcriptional repressor Whi5, which is an orthologue of the human tumor suppressor retinoblastoma protein (RB) [14], [15]. Upon its phosphorylation in late G1, Whi5 is released from its interaction with the SBF transcription factor, which is composed of the Swi4 DNA-binding protein and the Swi6 cofactor [16] and is orthologous to the human transcription factor E2F (see [14], [15]). Once free of Whi5, SBF can activate transcription of many genes required for progression into S phase, such as *CLN1* and -*2* that encode Cln1 and -2 [17], [18]. Cln3/Cdk1 also functions to activate the MBF transcription factor composed of the Mbp1 DNA-binding protein and Swi6 [19]. Unlike SBF, MBF remains bound to promoters and represses transcription outside of G1 phase; Cln3/Cdk1 is required to relieve this repression during G1 through an as yet undefined mechanism [20]. Many of the genes upregulated through MBF de-repression encode proteins involved in DNA replication and repair [21]. Nonetheless, several genes (including *CLN1*) are regulated by both SBF and MBF [22]. The consensus binding sites for SBF and MBF are referred to as the Swi4 cell cycle box (SCB) and MluI cell cycle box (MCB), respectively. For most direct targets of SBF or MBF, at least one copy of the corresponding cell cycle box sequence is found in the promoter region (see [22]).

Upon Cln3/Cdk1-mediated upregulation of *CLN1* and -*2* transcription, Cln1,-2/Cdk1 complexes assemble and directly catalyze phosphorylation of Sic1, leading to destruction of Sic1 through the ubiquitin-proteasome pathway [2], [23]–[27]. While Sic1 loss also occurs during meiosis, coincident with Clb5,-6/Cdk1 activation and S phase entry as during the cell cycle, the G1 cyclin/Cdk1 complexes do not function during meiosis [3], [28] and Sic1 loss does not depend on Cdk1 activity [29]. Proper timing of Sic1 destruction does, however, depend on Ime2 [3], [29]. This meiosis-specific protein kinase is required for optimal upregulation of many early meiotic genes and for normal progression through pre-meiotic DNA replication [30], [31]. It is also required for subsequent events in meiosis, such as expression of “middle” genes that regulate progression into the meiotic divisions [29], [30], [32]–[34]. An interesting theory based on the absence of Cln1,-2/Cdk1 activities during meiosis and the requirement of Ime2 for timely Sic1 destruction early in meiosis is that Ime2 directly replaces Cln1,-2/Cdk1 [3]. However, it is now known that while Sic1 destruction during meiosis requires the same Cdk1-targeted phosphorylation sites that operate during the cell cycle [4], [27], [35], [36], the Ime2 target specificity differs from that of Cdk1 [36]–[39]. An alternative hypothesis is that Ime2 indirectly promotes Sic1 phosphorylation by a distinct protein kinase, a possibility that we address in the work described here.

## Results

### Sic1 level decreases in *ime2Δ* cells

**Figure 1:**
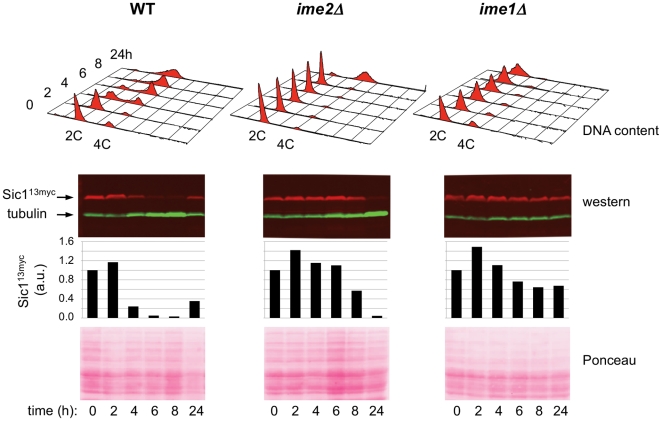
Sic1 steady-state levels. WT and indicated mutant cells were induced to enter meiosis in a synchronous fashion and followed through time (h = hours). DNA content was analyzed by flow cytometry to detect pre-meiotic DNA replication (2C to 4C transition). Sic1^13myc^ and tubulin were detected by western blotting. For each time point, Sic1^13myc^ level was quantified by determining the relative band intensities of Sic1^13myc^ (red) and tubulin (green) and normalizing the resulting Sic1^13myc^/tubulin ratio to the corresponding 0 hour ratio. Results are shown in graphical form (a.u., arbitrary units). Prior to immunodetection, membranes were stained with Ponceau S for total protein content assessment; regions that include Sic1^13myc^ and tubulin are shown. Strains used were YGB803, YGB787, and YGB804.

In wild type (WT) cells undergoing meiosis, Ime2 is required for the timely destruction of the B-type cyclin/CDK inhibitor Sic1, which leads to initiation of pre-meiotic DNA replication [3], [29]. In *ime2Δ* cells, pre-meiotic DNA replication is delayed, but not abolished [31]. To determine whether Sic1 is eventually degraded in *ime2Δ* cells to allow for delayed initiation of DNA replication, as has been suggested previously [36], we examined the behavior of epitope-tagged Sic1 (Sic1^13myc^) in WT and *ime2Δ* cells that were induced to enter meiosis synchronously. An *ime1Δ* strain was also included because cells lacking *IME1* cannot complete pre-meiotic DNA replication [31]. We analyzed DNA content by flow cytometry to evaluate DNA replication, and assessed the steady-state level of Sic1^13myc^ by western blotting (Fig. 1). As can be seen, Sic1^13myc^ began to disappear at the onset of DNA replication in WT cells. In *ime2Δ* cells, DNA replication occurred at a later stage, as expected, with concomitant Sic1 disappearance (see 24 hour time point). In contrast, neither DNA replication nor Sic1 disappearance was observed in *ime1Δ* cells within 24 hours. We conclude that Ime2 is not absolutely required for Sic1 destruction, suggesting that a distinct protein kinase is capable of catalyzing Sic1 phosphorylation.

### Ime2 affects expression of cell cycle box-containing genes

**Figure 2:**
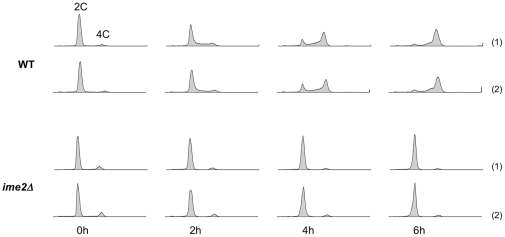
Time course for global gene expression analysis: cellular DNA content. WT and *ime2Δ* cells were induced to enter meiosis synchronously and harvested at regular time points for microarray analysis. DNA content was analyzed by flow cytometry to assess progression though early meiosis (DNA replication). Histograms for the biological replicates are shown. Strains used were DSY1089 and YGB221.

To further understand the role of Ime2 in promoting proper timing of pre-meiotic DNA replication, we compared global gene expression in WT and *ime2Δ* cells that were induced to enter meiosis. Our goal was to include analysis of early meiotic events before significant middle gene expression was induced, and so we compared cells at 0, 2, 4, and 6 hours after meiotic induction. To gauge progression through the early stages of meiosis, we measured DNA content by flow cytometry. The biological replicates were markedly similar by this criterion (Fig. 2). RNA was isolated from these cells and subjected to single color microarray analysis using the Agilent 60-mer oligonucleotide platform. Gene expression data resulting from our study are presented in Table S1. For most of our analyses, the 0 hour data served as the control values to which subsequent time point values were compared within an individual strain.

The expression data were first analyzed with T-Profiler, which scores the activities of defined gene sets [40]. Through T-profiler, the t-test is used to determine whether the mean expression of a group of genes is significantly different from the mean expression of all other genes in the microarray. The calculated t-values provide an indication of the degree of upregulation (t>0) or downregulation (t<0) for the particular comparison. To first validate our results within the context of the T-profiler approach, we compared our WT expression data with published meiotic expression data [41] generated from cells with the same genetic background as our cells (SK1 [42]). The expression patterns of various gene groups defined by consensus promoter motifs, based on t-values, correlated well with those that we observed in our experiment (Fig. S1A). We concluded that the T-profiler algorithm provided a suitable method to analyze gene expression in our study.

**Figure 3:**
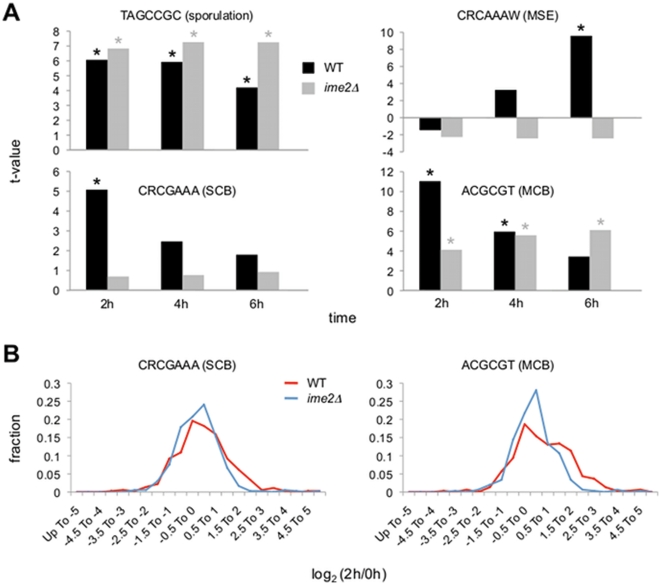
Analysis of consensus motif gene sets. *A*, Gene expression data from our time course were analyzed by T-profiler for average expression of gene groups defined by consensus promoter motifs. Results for selected gene groups characterized by the indicated sequences are shown (R = A or G; W = A or T). Comparisons were made between expression levels at 2, 4, and 6 h *v.* expression levels at 0 h. Asterisks indicate statistically significant values (E<0.05). *B*, Distributions of log_2_ (2 h/0 h) ratios for the CRCGAAA (left) and ACGCGT (right) gene sets are shown for WT and *ime2Δ* cells.

We next compared our WT and *ime2Δ* expression data, and results for specific gene groups as defined by consensus promoter motifs are shown in Fig. 3A and Table S2. Expression was compared at 2, 4, and 6 hours *v.* 0 hours for each strain. Certain sets, such as the “sporulation” group, exhibited robust average expression in both cell types. These data indicate that deletion of *IME2* did not indiscriminately prevent early meiotic gene expression as determined by T-profiler. In fact, the TAGCCGC sequence that defines this sporulation gene group is found in URS1 elements that initially act upstream of Ime2 in the meiotic transcriptional cascade (see [43]), and so this result would be expected. Furthermore, the slightly enhanced upregulation of this gene set for *ime2Δ* cells relative to WT cells, with increasing effect over time, might be explained by the fact that Ime2 negatively regulates its upstream activator Ime1 [30], [44], a protein that facilitates derepression of URS1 elements [45].

With other gene sets, we observed significant differences between WT and *ime2Δ* cells. It is known that normal middle gene expression depends on *IME2* [29], [30], [32]–[34], and we therefore anticipated differences in gene sets defined by middle sporulation elements (MSEs). As can be seen in Fig. 3A, the t-value of the indicated MSE set increased strikingly over time for WT cells, but remained low for *ime2Δ* cells throughout the experiment. Interestingly, two other gene groups also showed large differences in average expression, specifically at the early 2-hour time point (Fig. 3A). The t-values of the gene groups defined by the SCB or MCB promoter elements were significantly elevated (E<0.05) at 2 hours for WT cells, and then declined with time, indicative of early rather than middle expression. In *ime2Δ* cells, the SCB set showed little induction of average expression at any time point. By contrast, the MCB set showed significant upregulation at each time point in *ime2Δ* cells, but with a delayed pattern compared to WT cells: the degree of upregulation was much less at 2 hours relative to WT, and while upregulation increased modestly at 4 and 6 hours, it did not reach the magnitude of upregulation observed in WT cells at 2 hours. Note that the relative degree of maximal upregulation observed with the SCB and MCB groups in WT cells was similar during meiosis and the cell cycle as judged through T-profiler (Fig. S1B). Histograms comparing expression levels of the SCB and MCB groups at 2 *v.* 0 hours showed *IME2*-dependent differences in distribution, with WT sets skewed toward positive log_2_ ratio values relative to the *ime2Δ* sets (Fig. 3B). (Comparisons of cell cycle box element sets with all other genes in the microarray are shown in Fig. S2.) These profiles show that *IME2* status affected many genes in these two sets.

Focusing specifically on the 2 *v.* 0 hour expression data, we found that average expression of 8 of the 153 gene groups defined by consensus promoter motifs was significantly upregulated in WT cells (Table 1). Note that 4 of these 8 groups were defined by cell cycle box elements, and in 3 of these 4 cases the E-value for the corresponding *ime2Δ* expression data was equal to 1.0. In addition to analyzing expression relative to the 0-hour values, we directly compared expression values of WT cells with *ime2Δ* cells at each time point. The comparison at 2 hours revealed significant differences in average upregulation of 6 gene groups, 4 of which were defined by either SCB or MCB elements (Table 2). The other two groups were defined by sporulation-specific motifs that were also upregulated at 0 hours, unlike the cell cycle box groups. Taken together, these results demonstrate that Ime2 is required for normal upregulation of many genes that contain consensus SCB or MCB sites in their promoters.

**Table 1:** T-profiler analysis of consensus motifs for 2 *v.* 0 hours.

t-value	E-value	Mean log_2_ ratio						
Motif	Name^1^	WT	*ime2Δ*	WT	*ime2Δ*	WT	*ime2Δ*	ORFs
ACGCGT: **MCB**	MBP1	11.05	4.12	<1.0e-15	5.5e-03	0.566	0.149	299
TSGGCGGCTAWW	meiosis	7.81	9.89	8.4e-13	<1.0e-15	1.371	1.504	32
TCGGCGG	YAP5	6.97	8.00	4.6e-10	1.8e-13	0.726	0.722	81
GCGGCTA	sporulation	6.85	8.48	1.1e-09	<1.0e-15	0.618	0.668	104
TAGCCGC	sporulation	6.07	6.83	1.9e-07	1.2e-09	0.467	0.457	134
TKACGCGTT: **MCB**	MBP1	5.24	1.79	2.3e-05	1.0	0.956	0.246	29
CRCGAAA: **SCB**	SWI4	5.08	0.70	5.5e-05	1.0	0.205	−0.023	357
TTTCGCG: **SCB**	SWI4	3.84	−1.23	1.8e-02	1.0	0.238	−0.136	169

1 The element names shown here are those designated in T-profiler.

**Table 2:** T-profiler analysis of consensus motifs for WT *v. ime2Δ* at 2 hours.

Motif	Name	t-value	E-value	Mean log_2_ ratio	ORFS
ACGCGT: **MCB**	MBP1	12.49	<1.0e-15	0.522	299
CRCGAAA: **SCB**	SWI4	6.55	8.4e-09	0.272	357
TTTCGCG: **SCB**	SWI4	6.46	1.5e-08	0.380	169
GACACAA	sporulation	5.91	5.0e-07	0.387	137
TKACGCGTT: **MCB**	MBP1	5.21	2.8e-05	0.731	29
TTGTGTC	sporulation	4.14	5.1e-03	0.276	145

### Consensus motif discovery reveals that Ime2 is required for normal expression of genes containing the MCB promoter element

We next analyzed our data for consensus motifs associated with transcriptional upregulation through MatrixREDUCE [46] (see Table S3). This algorithm does not rely on defined gene groups but detects consensus sequences in promoters that are associated with up- or downregulation. Once again, expression was compared at 2, 4, and 6 hours *v.* 0 hours for each strain. Not surprisingly, CACAAAA, matching the MSE consensus sequence, was discovered as a significant element for WT cells at both 4 and 6 hours, but not at any time point for *ime2Δ* cells. In accordance with our previous results, ACGCGT, matching the MCB consensus sequence, was discovered as a significant element at both 2 and 4 hours for WT cells, but only at 6 hours for *ime2Δ* cells. We also compared WT with *ime2Δ* directly at each time point to discover sequences correlated to expression specifically in WT cells as opposed to *ime2Δ* cells. CACAAAA (MSE) was found to be significant specifically at 6 hours, while sequences closely related to the MCB consensus, ACGCG and CGCGTAA, were identified as significant motifs exclusively at 2 hours. In summary, these data are consistent with the well-established role of Ime2 upstream of the MSE element, and provide further evidence that Ime2 functions upstream of the MCB element. As was the case with analysis of a published meiotic time course of WT cells using a precursor algorithm named REDUCE [47], a consensus SCB motif was not detected from our WT data with MatrixREDUCE.

### Ime2 affects expression of genes that are targets of SBF or MBF during the cell cycle

Our results indicated that *IME2* operates upstream of genes containing consensus SCB or MCB elements, suggesting that Ime2 controls genes that respond to SBF or MBF during the cell cycle. To pursue the idea that SBF- and MBF-regulated genes could lie downstream of *IME2*, we turned to a recent investigation that served to characterize the transcriptional response of Cln3/Cdk1 during the cell cycle [22]. Several criteria were considered in this study, including gene expression, transcription factor occupancy, and consensus motif data, to identify those genes that are likely to be directly targeted by SBF or MBF in response to Cln3/Cdk1 activity.

**Figure 4:**
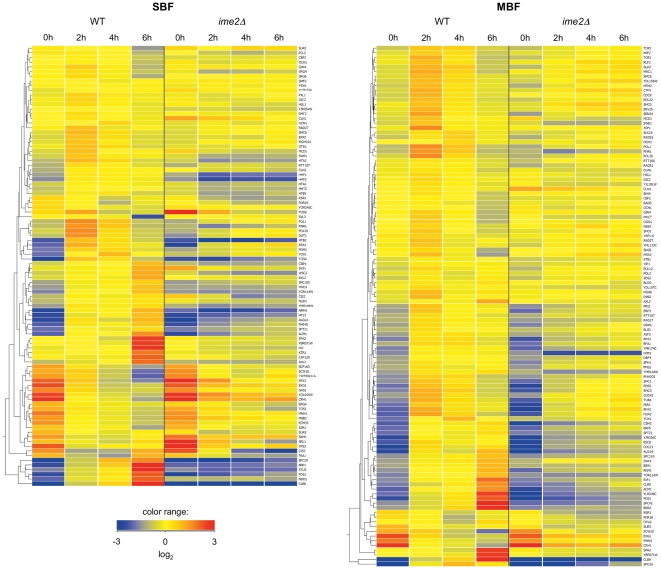
Expression of SBF and MBF targets in early meiosis. Gene-normalized hierarchical clustering analysis of MBF and SBF targets. For clustering procedure, see *Materials and Methods*.

**Figure 5:**
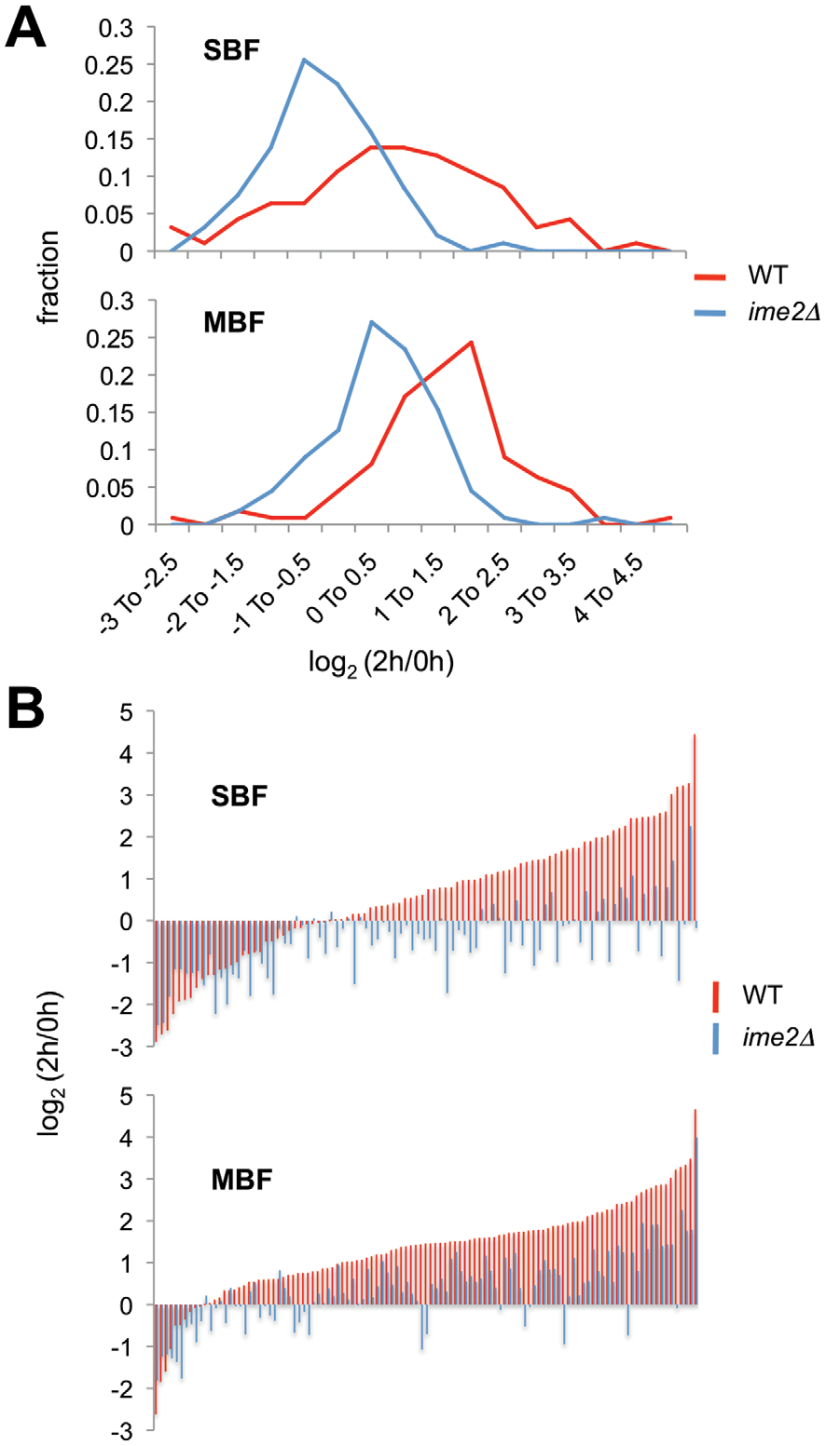
Examination of SBF and MBF targets at 2 hours. *A*, Distributions of log_2_ (2 h/0 h) ratios for SBF and MBF targets in WT and *ime2Δ* cells. *B*, Direct comparison of WT and *ime2Δ* log_2_ (2 h/0 h) ratios for each SBF and MBF target.

We performed cluster analyses on those genes that are induced by Cln3/Cdk1 and are considered targets of SBF (94 genes) or MBF (111 genes) (Fig. 4 and Table S4). (Note that these sets share 36 genes.) These clusters reveal a large number of genes in each group that were upregulated by 2 hrs in WT cells, with considerably different patterns in *ime2Δ* cells. These data were further analyzed specifically for expression changes from 0 to 2 hrs, shown as distributions of log_2_ ratios and as comparisons of log_2_ ratios for each gene (Fig. 5). For the SBF group, the mean log_2_ (2 hr/0 hr) values for WT and *ime2Δ* were 0.61 and −0.47, respectively, and for MBF they were 1.32 and 0.39, respectively. The WT and *ime2Δ* values were found to be significantly different for both the SBF and MBF sets (p<0.0001) as assessed by the two-tailed Wilcoxon signed-rank test (calculated *via* the VassarStats website at http://faculty.vassar.edu/lowry/wilcoxon.html). In summary, these data indicate that Ime2 is required for proper meiotic induction of many genes that respond to SBF or MBF during the cell cycle.

It is important to note that subsets of the SBF and MBF groups were not induced during meiosis in WT cells at 2 hours (see Figs. 4 and 5, and Table S4). This effect was more pronounced with the SBF targets. We did not detect enrichment in the repressed (log_2_ (2 h/0 h)<0) or induced (log_2_ (2 h/0 h)>0) SBF subsets for genes with known meiosis-related promoter elements; for the small subset of MBF targets that were repressed, we found enrichment for the MSE element and for targets that respond to both SBF and MBF during the cell cycle (see Table S5). Whether these characteristics are functionally important is not clear at this point. We further investigated the two SBF subsets for promoter motifs using the MUSA (Motif finding using an UnSupervised Approach) algorithm [48] (Table S6). Among the most significant promoter motifs discovered in the repressed subset was the CGAGAA & TTCTCG pair (P = 1.63e-06), which was not considered to be significant in the induced subset. On the other hand, the ATACATA & TATGTAT pair was discovered to be significant specifically in the induced subset (P = 9.68e-07). Further analysis will be required to determine whether these elements, or others, play a role in meiosis-specific expression of SBF targets. Regardless of mechanism, it is interesting that 9 of the 30 cell cycle-regulated SBF targets that are not induced at 2 hours during meiosis are involved in cell wall organization or biogenesis (determined through GO Slim Mapper at the *Saccharomyces* Genome Database (http://www.yeastgenome.org/) [49].

### Whi5 is not downstream of Ime2 in the DNA replication pathway

**Figure 6:**
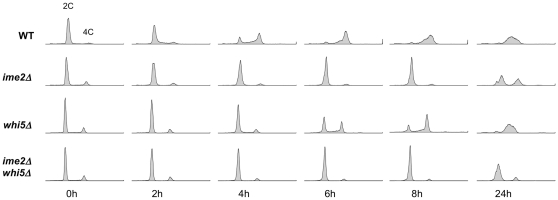
Epistasis analysis of *IME2* and *WHI5*. WT and mutant cells were induced to enter meiosis synchronously and DNA content was analyzed by flow cytometry at the indicated time points. This experiment was conducted in conjunction with the microarray experiment; therefore, sets of WT and *ime2Δ* histograms shown in Fig. 2 are reproduced here. Note that we have observed variability in the degree of DNA replication observed in *ime2Δ* cells at 24 h (*e.g.* compare with Fig. 1). Strains used were DSY1089, YGB221, YGB752, and YGB753.

Given the idea that an early Ime2 function operates through SBF and MBF, or meiotic versions of these factors, one possibility is that Ime2 behaves similarly to Cln3/Cdk1 by catalyzing phosphorylation of Whi5 to relieve its inhibitory effect (see [14], [15]). In such a scenario, Whi5 would lie just downstream of Ime2 in the pathway that ultimately leads to Sic1 destruction and replication origin firing. Epistasis analysis has revealed that deletion of *SIC1* accelerates initiation of pre-meiotic DNA replication in *ime2Δ* cells [3], and so we conducted a similar experiment in which we compared pre-meiotic DNA replication in *ime2Δ*, *whi5Δ*, and *ime2Δ whi5Δ* mutants. We found that deletion of *WHI5* alone delayed the onset of DNA replication relative to WT cells (Fig. 6). This result is consistent with published data showing that small cells exhibit delayed initiation of meiosis [50]. We also found that deletion of *WHI5* did not suppress, but appeared to enhance, the *ime2Δ*-associated delay in DNA replication initiation. This analysis suggests that *WHI5* is not downstream of *IME2*, and that Ime2 does not regulate pre-meiotic DNA replication by negatively regulating Whi5.

## Discussion

Ime2 protein kinase activity is required for proper progression through the early stages of meiosis [29]. However, critical substrates of this enzyme that specifically promote early meiotic progression have not been identified. The studies that we describe here were designed to define the position that Ime2 occupies in the pathway that leads to initiation of pre-meiotic DNA replication. This information will guide future studies aimed at characterizing mechanisms by which Ime2 directs early meiotic transitions.

We found that pre-meiotic DNA replication was delayed in *ime2Δ* cells, as previously reported [31], and demonstrated that Sic1 disappeared in this same window. It has been shown that Sic1 becomes ubiquitylated in *ime2Δ* cells, implying that Sic1 degradation occurs through the proteasome in the absence of Ime2 [36]. It has also been shown that delayed entry into pre-meiotic DNA replication in *ime2Δ* cells is accompanied by delayed DNA polymerase alpha-primase complex phosphorylation, which occurs during S phase in WT cells engaged in the mitotic cell cycle or undergoing meiosis [31], [51]. Therefore, by different criteria the delayed pre-meiotic DNA replication observed in *ime2Δ* cells resembles the WT process, suggesting that the normal mechanisms regulating DNA replication initiation occur in these cells, but with slower kinetics.

It is striking that the majority of Sic1 disappeared in *ime2Δ* cells by 24 hours. Previous studies have shown that the same phosphorylation sites control Sic1 stability during the cell cycle and meiosis [4], [27], [35], [36]. However, the Cln/Cdk1 enzymes do not operate during meiosis [3], [28]. While we cannot be certain at this point that destabilization of Sic1 in *ime2Δ* cells occurs through the same process as in WT cells, our results clearly indicate that the Sic1 steady state level can be decreased significantly in an Ime2-independent manner. Based on the phosphorylation sites that are required for Sic1 destruction during meiosis, we expect Sic1 phosphorylation to be catalyzed by a cyclin/CDK-like complex that normally requires Ime2 for activation but can eventually become activated in the absence of Ime2. Sic1 destabilization occurs in *IME2+* cells devoid of Clb5 and -6, or when Cdk1 is inhibited, indicating that Clb5,-6/Cdk1 or other Cdk1-containing complexes are not involved [29], [36]. Future work will be aimed at identifying the responsible protein kinase(s).

**Figure 7:**
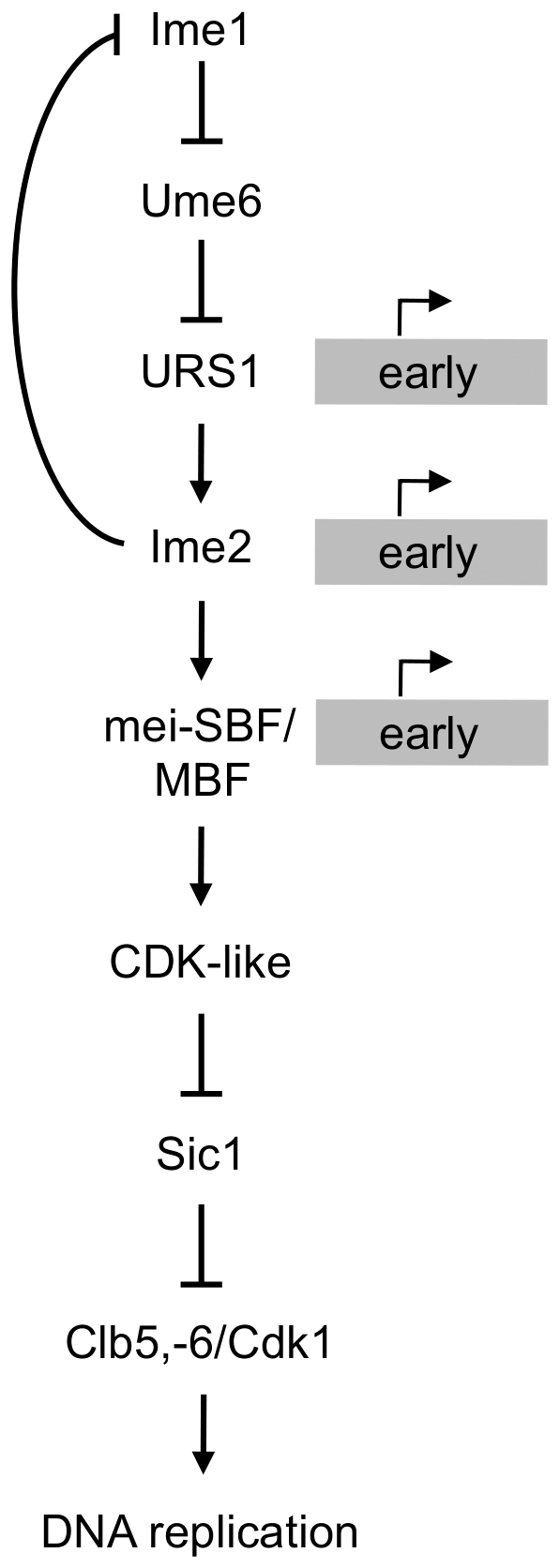
Model of early Ime2 function. Pathways are shown leading from Ime1 induction early in meiosis to DNA replication. Factors relevant to the discussion in the text are included. Ime1 activates early gene expression through de-repression at the URS1 element [45], which is found in many meiosis-specific promoters including that of *IME2* [43]. Ime2 in turn upregulates gene expression [43], and negatively affects Ime1 expression transcriptionally and post- transcriptionally [30], [44]. We hypothesize that Ime2 activation of the meiotic (mei) SBF/MBF transcriptional cascade leads to activation of a protein kinase with CDK specificity that enables Sic1 destruction, Clb5,-6/Cdk1 activation, and initiation of DNA replication.

Our examination of the Sic1 steady state level in *ime2Δ* cells suggests that Ime2 lies further upstream in promoting Sic1 destruction than originally suspected. Support for this hypothesis comes from our global gene expression analysis indicating that Ime2 activates expression of many genes that are controlled during the cell cycle by SBF or MBF. While expression of these genes was not abolished in the *ime2Δ* mutant, some redundancy could exist in controlling expression of these genes. This type of overlap has been well established in the mitotic cell cycle, as Bck2 induces transcription of many cell cycle-regulated genes regardless of cell cycle phase [52]. Such a secondary pathway might eventually allow for the delayed progression through pre-meiotic DNA replication observed in *ime2Δ* cells. From our demonstration that Ime2 significantly influences transcription of SBF and MBF targets, and from results that have been presented in the literature, we have arrived at the model depicted in Fig. 7. During the cell cycle, Cln3/Cdk1 lies upstream of SBF and MBF, while Cln1,-2/Cdk1 are downstream of Cln3/Cdk1 and respond to SBF and MBF [11]–[13], [17], [18]. Therefore, rather than serving simply as a meiotic substitute for Cln1,-2/Cdk1, it appears that Ime2 has an earlier function in the meiotic pathway, perhaps operating like Cln3/Cdk1 or acting even further upstream. In this position, Ime2 activity could induce an increased level of a gene product that influences Sic1 structure and stability directly, *e.g.* through Sic1 phosphorylation, or indirectly, *e.g.* through activation of the responsible Sic1 kinase.

Our data indicate that upregulation of SBF and MBF targets, while similar in the cell cycle and during meiosis, is not identical, as subsets that respond during the cell cycle are not induced during meiosis. It is important to consider that cell cycle box function during meiosis, including the nature of trans-acting factors that bind to these sequences, has not been defined on a general level. Mutation analysis has revealed that MCB elements do operate during meiosis, specifically with regard to the *CLB5* promoter [53]. However, this same study showed that Mbp1 is not required for normal meiotic upregulation of *CLB5*. By contrast, meiotic regulation of other genes with MCB promoter elements has been shown to involve MBF components: Mbp1 is required for proper upregulation of *RNR1* and *TMP1* [53], and Swi6 is required for proper upregulation of *RAD51* and *RAD54* [54]. (Note that *RAD54* is not included as a cell cycle-regulated MBF target in the data set that we used for our analyses [22].) The *CLB5* results suggest that meiotic versions of cell cycle box-binding factors could operate in some contexts. Strong support for this hypothesis comes from the report that cells lacking both Swi4 and Mbp1, which arrest in the mitotic cell cycle [19], can proceed through meiosis [3]. Regulatory subunits might also differ between meiosis and the cell cycle, given our evidence that Whi5 does not lie downstream of Ime2 in the pathway leading to pre-meiotic DNA replication. Regardless of the exact components, our data indicate that *IME2* acts during meiosis upstream of many genes controlled by SBF or MBF during the cell cycle. An important direction of our future research will be to precisely define the mechanism(s) by which Ime2 activity influences transcription of these genes.

## Materials and Methods

### Yeast strains

The diploid strains used in this study are listed in Table 3. *SIC1* was modified in a WT haploid to encode Sic1 with 13 myc epitope repeats at the C-terminus by PCR amplification and homologous recombination, as described [55]. Diploids containing this allele were then generated through standard techniques involving mating and sporulation with *ime2Δ::TRP1* [56] and *ime1Δ::natR* haploids. The *ime1Δ* haploid used for this purpose was generated from a WT haploid by homologous recombination using a PCR fragment amplified from genomic DNA of an *ime1Δ::kanMX4* deletion set mutant [57] (Open Biosystems). This marker was then switched to *natR*, as described [58]. *WHI5* was deleted in WT and *ime2Δ* haploids by homologous recombination using a PCR fragment amplified from the genomic DNA of a *whi5Δ::kanMX4* deletion set mutant [57] (Open Biosystems). Resulting haploids were then mated to generate the appropriate diploids. Deletions were verified through PCR analysis.

**Table 3:** Yeast strains used in this study.

Strain^1^	Relevant genotype	Ref.
YGB803	*MAT* **a**/*α ho::LYS2/″ lys2/″ ura3/″ leu2::hisG/″ trp1::hisG/″ arg4-BglII/arg4-NspI his4B/his4X SIC1^13myc^::kanMX6/SIC1*	this study
YGB787	*MAT* **a**/*α ho::LYS2/″ lys2/″ ura3/″ leu2::hisG/″ trp1::hisG/″ arg4-BglII/arg4-NspI his4B/his4X SIC1^13myc^::kanMX6/SIC1 ime2Δ::TRP1/″*	this study
YGB804	*MAT* **a**/*α ho::LYS2/″ lys2/″ ura3/″ leu2::hisG/″ trp1::hisG/″ arg4-BglII/arg4-NspI his4B/his4X SIC1^13myc^::kanMX6/SIC1 ime1Δ::nat^R^/″*	this study
DSY1089	*MAT* **a**/*α ho::LYS2/″ lys2/″ ura3/″ leu2::hisG/″ trp1::hisG/″ arg4-BglII/arg4-NspI his4B/his4X*	[4]
YGB221	*MAT* **a**/*α ho::LYS2/″ lys2/″ ura3/″ leu2::hisG/″ trp1::hisG/″ arg4-BglII/arg4-NspI his4B/his4X ime2Δ::TRP1/″*	[56]
YGB752	*MAT* **a**/*α ho::LYS2/″ lys2/″ ura3/″ leu2::hisG/″ trp1::hisG/″ arg4-BglII/arg4-NspI his4B/his4X whi5Δ::kanMX4/″*	this study
YGB753	*MAT* **a**/*α ho::LYS2/″ lys2/″ ura3/″ leu2::hisG/″ trp1::hisG/″ arg4-BglII/arg4-NspI his4B/his4X ime2Δ::TRP1/″ whi5Δ::kanMX4/″*	this study

1 Strains are congenic with SK1 [42] and are listed in the order that they appear in the text.

### Induction of meiosis

Cells were treated to enter meiosis synchronously based on an established procedure [56], [59], briefly described here: Cells were streaked onto YPG plates (1% yeast extract, 2% peptone, 3% glycerol, 2% agar) and incubated for 3–4 days (all incubations at 30°C). Single colonies were then inoculated into YPD (1% yeast extract, 2% peptone, 2% dextrose (glucose)) and incubated overnight. The cells were then inoculated into YPA (1% yeast extract, 2% peptone, 2% potassium acetate) at a starting OD_600_ of ∼0.1. The cells were incubated for 16 hrs, washed once with SPM (0.3% potassium acetate, 0.02% raffinose, supplements for auxotrophies) and then resuspended with SPM. The OD_600_ of the final SPM suspensions was adjusted to ∼1 in most of our experiments. Aliquots of cells were harvested immediately (0 hours) and at regular time points after incubation at 30°C for DNA content analysis (stored in 70% ethanol at 4°C), RNA analysis (stored at −80°C), and protein analysis (stored at −70°C).

### DNA content analysis

Cells that had been fixed in 70% ethanol were treated with RNAse and then proteinase K, and stained with SYBR Green I DNA binding dye (Invitrogen). The cells were then analyzed by flow cytometry using a BD FACSCantoII system (Microscopy, Imaging and Cytometry Resources Core at Karmanos Cancer Institute, Wayne State University). DNA content histograms were generated using WinMDI software (developed by Joseph Trotter at the Scripps Research Institute).

### Protein analysis

Whole cell extracts were generated by alkali treatment and boiling [60]. Steady-state levels of Sic1^13myc^ were examined by western blotting. Nitrocellulose-bound proteins were first stained with Ponceau S to assess general protein levels. Immunostaining was then conducted using a mouse monoclonal primary antibody directed against the myc epitope (clone 9E10, Santa Cruz Biotechnology) followed by Alexa Fluor 680-conjugated goat anti-mouse IgG (Molecular Probes) secondary antibody. Simultaneously, we analyzed tubulin using a rat monoclonal primary antibody (clone YOL 1/34; Serotec) followed by IRDye 800-conjugated goat anti-rat IgG (Rockland) secondary antibody. Bands were visualized and band intensities were quantified with a LI-COR Odyssey infrared imaging system and associated software.

### Microarray analysis

Total RNA was isolated from harvested cells using a hot phenol procedure [61] and checked for adequate quality with an Agilent 2100 Bioanalyzer (Agilent Technologies). Aminoallyl-aRNA was then generated using the TargetAMP 1-Round Aminoallyl-aRNA Amplification Kit 101 (Epicentre) and purified using the RNeasy Mini Kit (Qiagen). The aminoallyl-aRNA was incubated with Alexa Fluor Reactive Dye Alexa 555 (Molecular Probes) and purified through another RNeasy column to remove all of the unincorporated dye. Hybridization was conducted with the Agilent 60-mer oligo microarray (Yeast Oligo Microarray 8×15 K) in Agilent SureHyb hybridization chambers. After hybridization, the slides were washed following the recommended Agilent protocol. The slides were immediately scanned with an Agilent dual laser scanner, with the photo multiplier tube set to an extended dynamic range for the green channel (High 100% and Low 10%). Tiff images were analyzed using Agilent's feature extraction software FE 10.7.1.1 to obtain fluorescent intensities for each spot on the arrays.

Microarray data were imported into GeneSpring version 11.0.2 for normalization and data analysis. Gene expression values (indicated as log_2_) were normalized on each microarray using the median signal intensity of the array. Replicate microarray data were used to perform statistical analyses for gene expression levels at each time point. The data are MIAME compliant and have been deposited into the Gene Expression Omnibus (GEO) database (http://www.ncbi.nlm.nih.gov/geo/), series identifier GSE26649. Consensus motif analysis was performed using the T-Profiler tool (http://www.t-profiler.org/) [40] and MatrixREDUCE [46] (default settings) developed by Harmen Bussemaker and colleagues (Columbia University), and the MUSA algorithm [48] available through the Yeastract website (http://www.yeastract.com) (see [62]). With the exception of the validation analysis presented in Fig. S1A, we did not include expression data for *IME2* or *TRP1* because *IME2* is specifically present in our WT cells and *TRP1* is specifically present in our *ime2Δ* cells. For hierarchical clustering analysis, we employed gene-normalized log_2_ values. Individual array-based expression values (16 per gene including both WT and *ime2Δ*) were first normalized to the median WT expression value. The normalized replicate values were then averaged. Clustering was performed on the WT data using Manhattan distance (city block) with average linkage, and the *ime2Δ* heat map was created based on the WT gene order.

## Supporting Information

Figure S1 **Consensus motif analysis of available meiosis and cell cycle data.** Data from WT cells were analyzed by T-profiler for average expression of gene groups defined by the indicated consensus motifs. *A*, top, meiotic time course study in which expression at indicated time points was compared with expression prior to meiotic entry [41]; bottom, our WT time course (in this case including expression data for *IME2* and *TRP1*). *B*, cell cycle study involving release from alpha factor-induced G1 arrest; gene expression at indicated time points was compared with expression in an asynchronous population [63].
(TIF)

Figure S2 **Distributions of SCB and MCB gene sets versus all other genes.** Distributions of log_2_ (2 h/0 h) ratios for CRCGAAA and non-CRCGAAA genes (left) and for ACGCGT and non-ACGCGT genes (right) are shown for WT (upper) and *ime2Δ* (lower) cells.
(TIF)

Table S1 **Microarray data.** Gene expression values were normalized to the median intensity of each array. The resulting log_2_ values are shown. Means and standard deviations of the replicate values are also shown.
(XLSX)

Table S2 **T-Profiler consensus motif analysis.** Data are shown for comparison of 2, 4, and 6 hours with 0 hours for both cell types (a–f) and for comparison of the two cell types at each time point (g–j). Only gene groups considered statistically significant (E<0.05) are shown (see [40]); red = upregulation, green = downregulation.
(XLSX)

Table S3 **MatrixREDUCE consensus motif analysis.** Data are shown for comparison of 2, 4, and 6 hours with 0 hours for both cell types (a–f) and for comparison of the two cell types at each time point (g–j). The P-value provides an indication of confidence in the motif, which is calculated based on a t-value derived from an associated regression coefficient (F-value) (see [46]); red = upregulation, green = downregulation.
(XLSX)

Table S4 **Gene-normalized hierarchical clustering of SBF and MBF targets.** The log_2_ values shown in this table were used to generate the heat maps shown in Fig. 4.
(XLSX)

Table S5 **Overlap of SBF and MBF sets with known meiosis-related gene sets.** Gene sets defined as SBF or MBF targets during the cell cycle were compared with gene sets defined by meiosis-related promoter elements to determine the degree of overlap. Numbers of genes in each set are shown, with “Both” indicating those genes that both respond to the cell cycle regulator and contain the indicated meiosis-related element. Statistical analysis to determine whether meiosis-related elements were differentially represented in the repressed (log_2_ (2 h/0 h)<0) or induced (log_2_ (2 h/0 h)>0) subsets of SBF or MBF target sets is shown in the bottom table (restricted to cases in which the “Both” category described above is >4). Further analysis includes comparison of subsets that are targeted by only SBF or MBF during the cell cycle with those that are targeted by both. R = A or G, W = A or T.
(XLSX)

Table S6 **Consensus motif analysis of SBF targets.** The repressed (log_2_ (2 h/0 h)<0) and induced (log_2_ (2 h/0 h)>0) subsets of the SBF target gene set were analyzed for consensus promoter motifs through the MUSA algorithm [48]. Motifs are shown, along with the number of genes in the set (quorum) that contain this motif. The P-value indicates the probability that the motif would occur by chance, and motifs for which P<0.001 are shown.
(XLSX)
