# Supplementary figures and images for: Yeast IME2 Functions Early in Meiosis Upstream of Cell Cycle-Regulated SBF and MBF Targets
Source: PLoS One. 2012 Feb 29;7(2):e31575. doi: 10.1371/journal.pone.0031575 (PMC3290606; doi:10.1371/journal.pone.0031575)

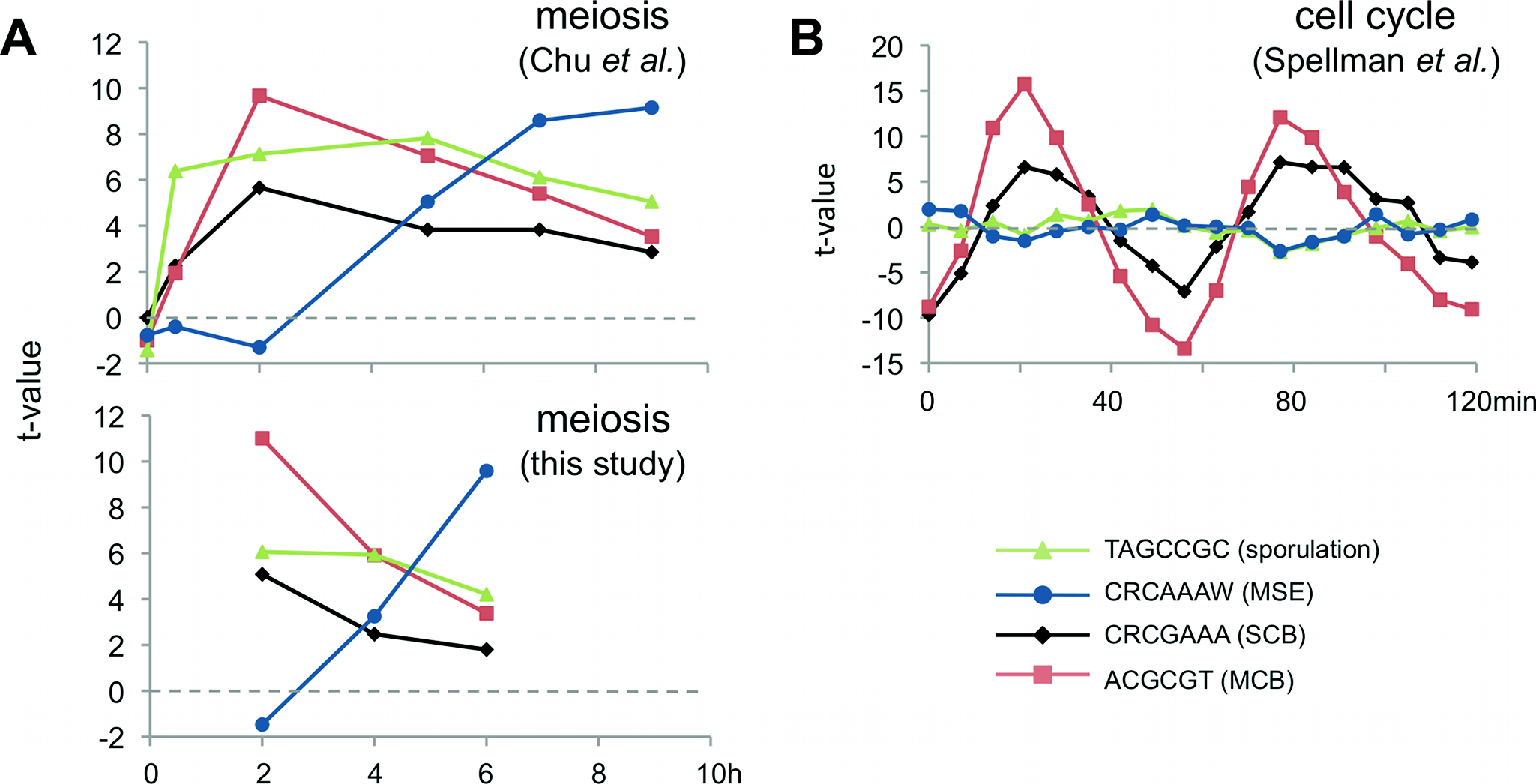

Supplement: Figure S1 — Consensus motif analysis of available meiosis and cell cycle data. Data from WT cells were analyzed by T-profiler for average expression of gene groups defined by the indicated consensus motifs. A, top, meiotic time course study in which expression at indicated time points was compared with expression prior to meiotic entry [41]; bottom, our WT time course (in this case including expression data for IME2 and TRP1). B, cell cycle study involving release from alpha factor-induced G1 arrest; gene expression at indicated time points was compared with expression in an asynchronous population [63]. (TIF) [file pone.0031575.s001.tif]

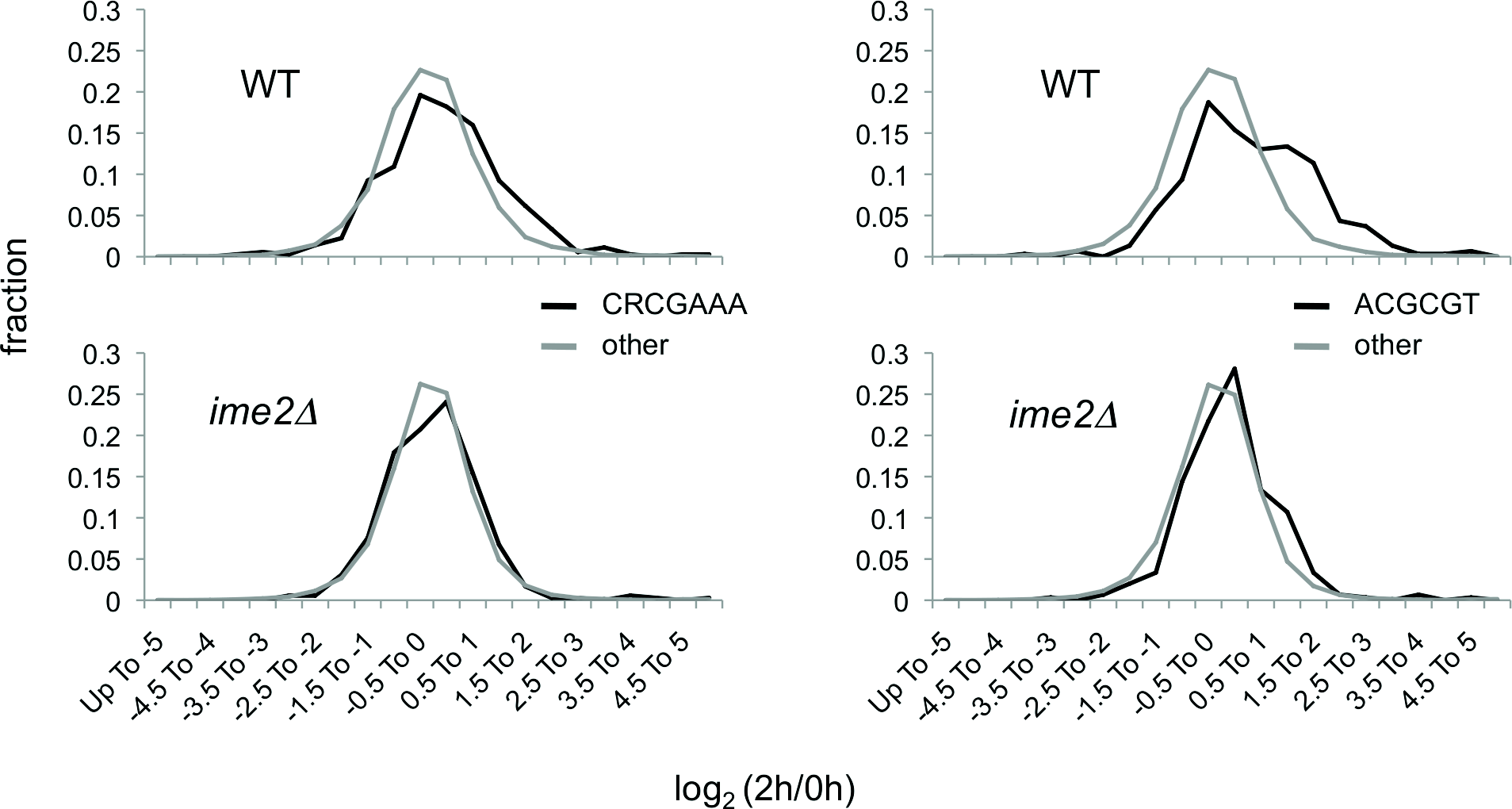

Supplement: Figure S2 — Distributions of SCB and MCB gene sets versus all other genes. Distributions of log2 (2 h/0 h) ratios for CRCGAAA and non-CRCGAAA genes (left) and for ACGCGT and non-ACGCGT genes (right) are shown for WT (upper) and ime2Δ (lower) cells. (TIF) [file pone.0031575.s002.tif]
